# Bis(2,3-dimethyl­butane-2,3-diamine)nickel(II) dinitrate monohydrate

**DOI:** 10.1107/S1600536809011702

**Published:** 2009-04-02

**Authors:** Shen-Xin Li, Li-Ke Zou, Bin Xie, Jun Wang, Jian-Zhang Li

**Affiliations:** a School of Chemistry & Pharmaceutical Engineering, Sichuan University of Science & Engineering, Zigong, Sichuan 643000, People’s Republic of China

## Abstract

In the title compound, [Ni(C_6_H_16_N_2_)_2_](NO_3_)_2_·H_2_O, the bis­(2,3-dimethyl­butane-2,3-diamine)nickel(II) complex cation possesses a relatively undistorted square-planar geometry about the Ni atom, which lies on an inversion centre and is coordinated by four N atoms from two symmetry-related 2,3-diamino-2,3-dimethyl­butane (tmen) ligands. The amine groups are N—H⋯O hydrogen bonded to the nitrate anions, which are, in turn, linked by inter­stitial water mol­ecules lying on a twofold axis. The infinite zigzag chains thus formed along [001] are further connected to each other by N—H⋯O hydrogen bonds towards the water mol­ecules, forming layers of two-dimensional hydrogen-bonded arrays.

## Related literature

For general background, see: Cheng *et al.* (2002 ▶). For related structures, see: Aranda *et al.* (1977 ▶); Beltran *et al.* (1978 ▶). For bond-length data, see Allen *et al.* (1987 ▶).
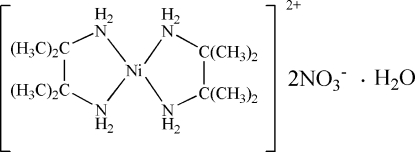


## Experimental

### 

#### Crystal data

[Ni(C_6_H_16_N_2_)_2_](NO_3_)_2_·H_2_O
*M* *_r_* = 433.14
Monoclinic, 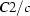

*a* = 21.788 (3) Å
*b* = 7.892 (3) Å
*c* = 13.997 (4) Å
β = 121.26 (3)°
*V* = 2057.4 (12) Å^3^
*Z* = 4
Mo *K*α radiation
μ = 0.99 mm^−1^
*T* = 292 K
0.50 × 0.46 × 0.40 mm

#### Data collection

Enraf–Nonius CAD-4 diffractometer
Absorption correction: spherical (*PLATON*; Spek, 2009 ▶) *T*_min_ = 0.638, *T*_max_ = 0.694
2099 measured reflections
1895 independent reflections
1314 reflections with *I* > 2σ(*I*)
*R*_int_ = 0.020
3 standard reflections every 100 reflections intensity decay: 0.8%

#### Refinement

*R*[*F* ^2^ > 2σ(*F* ^2^)] = 0.054
*wR*(*F* ^2^) = 0.165
*S* = 1.09
1895 reflections
129 parameters
1 restraint
H atoms treated by a mixture of independent and constrained refinement
Δρ_max_ = 0.96 e Å^−3^
Δρ_min_ = −0.65 e Å^−3^

### 

Data collection: *CAD-4 Software* (Enraf–Nonius, 1989 ▶); cell refinement: *CAD-4 Software*; data reduction: *XCAD4* (Harms & Wocadlo, 1995 ▶); program(s) used to solve structure: *SHELXS97* (Sheldrick, 2008 ▶); program(s) used to refine structure: *SHELXL97* (Sheldrick, 2008 ▶); molecular graphics: *ORTEP-3 for Windows* (Farrugia, 1997 ▶) and *DIAMOND* (Brandenburg & Putz, 2005 ▶); software used to prepare material for publication: *SHELXL97*.

## Supplementary Material

Crystal structure: contains datablocks I, global. DOI: 10.1107/S1600536809011702/zl2188sup1.cif

Structure factors: contains datablocks I. DOI: 10.1107/S1600536809011702/zl2188Isup2.hkl

Additional supplementary materials:  crystallographic information; 3D view; checkCIF report

## Figures and Tables

**Table 1:** Hydrogen-bond geometry (Å, °)

*D*—H⋯*A*	*D*—H	H⋯*A*	*D*⋯*A*	*D*—H⋯*A*
N1—H1*A*⋯O3^i^	0.90	2.17	3.048 (7)	166
N1—H1*B*⋯O*W*^ii^	0.90	2.23	3.120 (5)	170
N2—H2*A*⋯O2^iii^	0.90	2.09	2.936 (7)	157
O*W*—H*W*1⋯O1	0.91 (11)	1.95 (11)	2.824 (6)	160 (12)
O*W*—H*W*1⋯O2	0.91 (11)	2.57 (11)	3.106 (8)	118 (9)

Symmetry codes: (i) 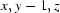; (ii) 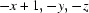; (iii) 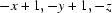.

**Fig. 1.**
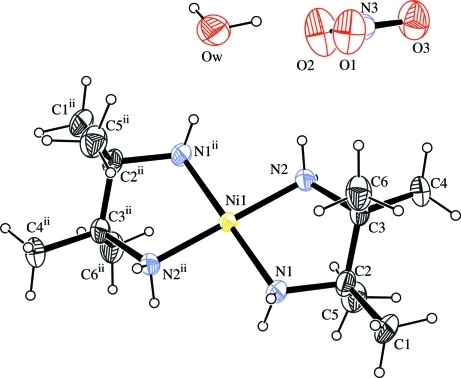
A view of the title compound showing the atom-labelling scheme. Displacement ellipsoids are drawn at the 20% probability level. H atoms are represented as small spheres of arbitrary radii. [Symmetry code: (ii) -x + 1,-y, -z].

**Fig. 2.**
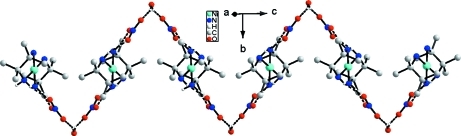
A view of the zigzag chain with hydrogen bonds shown as dashed lines, H atoms on the C atoms have been omitted for the sake of clarity.

**Fig. 3.**
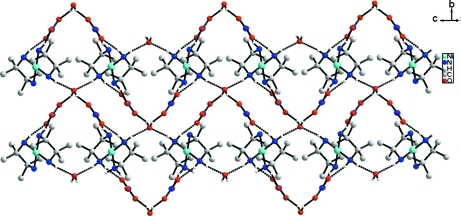
A view of the two-dimensional hydrogen bonded array with hydrogen bonds shown as dashed lines. H atoms on the C atoms have been omitted for the sake of clarity.

## supplementary crystallographic information

### Comment

The crystal structures of [Ni(tmen)_2_]Cl_2_.2H_2_O and [Ni(tmen)_2_](tca)_2_ (where tmen is 2,3-diamino-2,3-dimethylbutane, tca is tricloroacetate) have been described by Aranda *et al.* (1977) and Beltran *et al.* (1978) respectively. Our interest into the Ni and Co complexs of tmen is based on their potential use as efficient mimic models of natural enzymes for phosphate hydrolysis (Cheng *et al.* 2002). In this work the crystal structure of the title molecule [Ni(tmen)_2_](NO_3_)_2_.H_2_O is reported.

In the title compound, the Ni^II^ atom exhibits a relatively undistorted square-planar geometry (Fig.1), which lies on an inversion centre and is coordinated by four N atoms from two tmen ligands, with Ni—N interatomic distances of 1.890 (3)–1.898 (3) Å and N—Ni—N bond angles of 85.56 (14)–94.44 (14)°. All the other bond lengths and angles in the complex are generally within normal ranges (Allen *et al.*, 1987).

A striking feature of this compound resides in its zigzag chain structure formed through hydrogen bonds, with a solvate water molecule lying on a two fold axis, as depicted in Fig.2. The amine groups are N—H···O hydrogen bonding to the nitrate anions which are in turn linked by interstitial water molecules. The zigzag structure is composed of (tmen ligand) N—H···O (nitrate anion) and (water molecule) O—H···O (nitrate anion) hydrogen bonds (Table 1). The N—H···O distances for the hydrogen bonding of the tmen ligand and the nitrate anion range from 2.936 (7) to 3.048 (7) Å in the chain. Both O—H···O hydrogen bonds for the uncoordinated water molecule are 2.824 (6) Å. The thus formed infinite zigzag chains along [001] are further connected with each other by N—H···O hydrogen bonds towards the water molecules to form layers of two-dimensional hydrogen bonded arrays, as shown in Fig.3.

### Experimental

2,3-Diamino-2,3-dimethylbutane (tmen) (0.232 g, 2 mmol) and Ni(NO_3_)_2_.6H_2_O (0.291 g, 1 mmol) were dissolved in 20 ml distilled water, the solution was filtrated and the filtrate was kept at room temperature for six months after which yellow to green crystals suitable for X-ray diffraction studies were obtained, yield 37%. Selected infrared spectral (KBr) data (cm^-1^): ν[O—H] = 3399.9, ν[N—H] = 3187.1 and 3093.7, ν[N—O] = 1384.8, δ[N—H] = 1601.2.

### Refinement

H atoms on C and N atoms were fixed geometrically and constrained to ride on their parent atoms, with C—H = 0.96 Å (methyl) and N—H = 0.90 Å, and with *U*_iso_(H) = 1.2*U*_eq_(N) or *U*_iso_(H) = 1.5*U*_eq_(C). The water H atoms were determined with difference Fourier syntheses and refined isotropically.

### Crystal data

**Table:**

[Ni(C_6_H_16_N_2_)_2_](NO_3_)_2_·H_2_O	*F*(000) = 928
*M**_r_* = 433.14	*D*_x_ = 1.398 Mg m^−^^3^
Monoclinic, *C*2/*c*	Mo *K*α radiation, λ = 0.71073 Å
Hall symbol: -C 2yc	Cell parameters from 20 reflections
*a* = 21.788 (3) Å	θ = 4.4–7.1°
*b* = 7.892 (3) Å	µ = 0.99 mm^−^^1^
*c* = 13.997 (4) Å	*T* = 292 K
β = 121.26 (3)°	Block, yellow-green
*V* = 2057.4 (12) Å^3^	0.50 × 0.46 × 0.40 mm
*Z* = 4	

### Data collection

**Table:**

Enraf–Nonius CAD-4 diffractometer	1314 reflections with *I* > 2σ(*I*)
Radiation source: fine-focus sealed tube	*R*_int_ = 0.020
graphite	θ_max_ = 25.5°, θ_min_ = 2.2°
ω/2θ scans	*h* = −26→22
Absorption correction: for a sphere (*PLATON*; Spek, 2009)	*k* = −3→9
*T*_min_ = 0.638, *T*_max_ = 0.694	*l* = −16→16
2099 measured reflections	3 standard reflections every 100 reflections
1895 independent reflections	intensity decay: 0.8%

### Refinement

**Table:**

Refinement on *F*^2^	Secondary atom site location: difference Fourier map
Least-squares matrix: full	Hydrogen site location: mixed
*R*[*F*^2^ > 2σ(*F*^2^)] = 0.054	H atoms treated by a mixture of independent and constrained refinement
*wR*(*F*^2^) = 0.165	*w* = 1/[σ^2^(*F*_o_^2^) + (0.0951*P*)^2^ + 1.294*P*] where *P* = (*F*_o_^2^ + 2*F*_c_^2^)/3
*S* = 1.09	(Δ/σ)_max_ < 0.001
1895 reflections	Δρ_max_ = 0.96 e Å^−^^3^
129 parameters	Δρ_min_ = −0.65 e Å^−^^3^
1 restraint	Extinction correction: *SHELXL97* (Sheldrick, 2008), Fc^*^=kFc[1+0.001xFc^2^λ^3^/sin(2θ)]^-1/4^
Primary atom site location: structure-invariant direct methods	Extinction coefficient: 0.0084 (13)

### Special details

**Table:**

Geometry. All e.s.d.'s (except the e.s.d. in the dihedral angle between two l.s. planes) are estimated using the full covariance matrix. The cell e.s.d.'s are taken into account individually in the estimation of e.s.d.'s in distances, angles and torsion angles; correlations between e.s.d.'s in cell parameters are only used when they are defined by crystal symmetry. An approximate (isotropic) treatment of cell e.s.d.'s is used for estimating e.s.d.'s involving l.s. planes.
Refinement. Refinement of *F*^2^ against ALL reflections. The weighted *R*-factor *wR* and goodness of fit *S* are based on *F*^2^, conventional *R*-factors *R* are based on *F*, with *F* set to zero for negative *F*^2^. The threshold expression of *F*^2^ > σ(*F*^2^) is used only for calculating *R*-factors(gt) *etc*. and is not relevant to the choice of reflections for refinement. *R*-factors based on *F*^2^ are statistically about twice as large as those based on *F*, and *R*- factors based on ALL data will be even larger.

### Fractional atomic coordinates and isotropic or equivalent isotropic displacement parameters (Å^2^)

**Table:**

	*x*	*y*	*z*	*U*_iso_*/*U*_eq_	
Ni1	0.5000	0.0000	0.0000	0.0472 (3)	
N1	0.57151 (17)	−0.1127 (5)	−0.0139 (3)	0.0616 (10)	
H1A	0.5944	−0.1869	0.0428	0.074*	
H1B	0.5503	−0.1720	−0.0781	0.074*	
N2	0.56062 (17)	0.1927 (5)	0.0399 (3)	0.0605 (9)	
H2A	0.5415	0.2668	−0.0172	0.073*	
H2B	0.5629	0.2438	0.0992	0.073*	
C1	0.6937 (2)	−0.0917 (8)	0.0195 (5)	0.0874 (17)	
H1C	0.7082	−0.1557	0.0863	0.131*	
H1D	0.7311	−0.0134	0.0328	0.131*	
H1E	0.6851	−0.1674	−0.0399	0.131*	
C2	0.6254 (2)	0.0057 (6)	−0.0131 (4)	0.0668 (12)	
C3	0.6356 (2)	0.1470 (6)	0.0685 (4)	0.0668 (13)	
C4	0.6736 (3)	0.3029 (7)	0.0615 (5)	0.0790 (14)	
H4A	0.7193	0.2710	0.0725	0.119*	
H4B	0.6805	0.3822	0.1182	0.119*	
H4C	0.6449	0.3543	−0.0108	0.119*	
C5	0.5928 (3)	0.0831 (9)	−0.1304 (4)	0.0931 (17)	
H5A	0.5753	−0.0059	−0.1851	0.140*	
H5B	0.6288	0.1469	−0.1345	0.140*	
H5C	0.5538	0.1566	−0.1448	0.140*	
C6	0.6758 (3)	0.0776 (9)	0.1912 (4)	0.0881 (17)	
H6A	0.6539	−0.0264	0.1937	0.132*	
H6B	0.6729	0.1594	0.2395	0.132*	
H6C	0.7253	0.0574	0.2155	0.132*	
O1	0.6199 (3)	0.4468 (7)	0.2673 (5)	0.1304 (19)	
O2	0.5352 (3)	0.5668 (9)	0.1343 (6)	0.163 (3)	
O3	0.6359 (3)	0.6686 (7)	0.1958 (5)	0.1408 (19)	
N3	0.5972 (3)	0.5628 (7)	0.1999 (5)	0.0856 (13)	
OW	0.5000	0.2793 (8)	0.2500	0.115 (2)	
HW1	0.540 (4)	0.342 (12)	0.271 (10)	0.26 (6)*	

### Atomic displacement parameters (Å^2^)

**Table:**

	*U*^11^	*U*^22^	*U*^33^	*U*^12^	*U*^13^	*U*^23^
Ni1	0.0355 (4)	0.0458 (4)	0.0616 (5)	−0.0018 (3)	0.0261 (3)	−0.0082 (4)
N1	0.0446 (18)	0.061 (2)	0.085 (3)	0.0038 (16)	0.0371 (18)	−0.0057 (19)
N2	0.0505 (19)	0.0500 (19)	0.085 (3)	−0.0051 (15)	0.0379 (19)	−0.0108 (19)
C1	0.051 (3)	0.102 (4)	0.120 (5)	0.010 (3)	0.053 (3)	−0.003 (4)
C2	0.047 (2)	0.081 (3)	0.080 (3)	−0.005 (2)	0.038 (2)	−0.001 (3)
C3	0.043 (2)	0.073 (3)	0.084 (3)	−0.010 (2)	0.033 (2)	−0.009 (3)
C4	0.063 (3)	0.080 (3)	0.095 (4)	−0.023 (3)	0.042 (3)	−0.001 (3)
C5	0.087 (4)	0.125 (5)	0.071 (3)	−0.017 (4)	0.044 (3)	0.001 (4)
C6	0.062 (3)	0.120 (5)	0.066 (3)	−0.016 (3)	0.022 (3)	0.006 (3)
O1	0.097 (3)	0.123 (4)	0.154 (5)	0.028 (3)	0.053 (3)	0.068 (4)
O2	0.104 (4)	0.145 (4)	0.174 (6)	0.009 (4)	0.024 (4)	0.071 (4)
O3	0.143 (4)	0.101 (4)	0.188 (6)	−0.010 (3)	0.093 (4)	0.025 (4)
N3	0.087 (3)	0.061 (3)	0.117 (4)	0.012 (3)	0.059 (3)	0.000 (3)
OW	0.151 (7)	0.076 (4)	0.142 (6)	0.000	0.093 (6)	0.000

### Geometric parameters (Å, °)

**Table:**

Ni1—N1	1.890 (3)	C3—C4	1.513 (6)
Ni1—N1^i^	1.890 (3)	C3—C6	1.567 (7)
Ni1—N2	1.898 (3)	C4—H4A	0.9600
Ni1—N2^i^	1.898 (3)	C4—H4B	0.9600
N1—C2	1.496 (5)	C4—H4C	0.9600
N1—H1A	0.9000	C5—H5A	0.9600
N1—H1B	0.9000	C5—H5B	0.9600
N2—C3	1.510 (5)	C5—H5C	0.9600
N2—H2A	0.9000	C6—H6A	0.9600
N2—H2B	0.9000	C6—H6B	0.9600
C1—C2	1.520 (6)	C6—H6C	0.9600
C1—H1C	0.9600	O1—N3	1.220 (7)
C1—H1D	0.9600	O2—N3	1.176 (6)
C1—H1E	0.9600	O3—N3	1.209 (6)
C2—C3	1.528 (7)	OW—HW1	0.91 (11)
C2—C5	1.537 (7)		
			
N1—Ni1—N1^i^	180.0	C3—C2—C5	108.4 (4)
N1—Ni1—N2	85.56 (14)	N2—C3—C4	110.0 (4)
N1^i^—Ni1—N2	94.44 (14)	N2—C3—C2	105.0 (3)
N1—Ni1—N2^i^	94.44 (14)	C4—C3—C2	114.7 (4)
N1^i^—Ni1—N2^i^	85.56 (14)	N2—C3—C6	106.6 (4)
N2—Ni1—N2^i^	180.0	C4—C3—C6	110.0 (4)
C2—N1—Ni1	113.0 (3)	C2—C3—C6	110.2 (4)
C2—N1—H1A	109.0	C3—C4—H4A	109.5
Ni1—N1—H1A	109.0	C3—C4—H4B	109.5
C2—N1—H1B	109.0	H4A—C4—H4B	109.5
Ni1—N1—H1B	109.0	C3—C4—H4C	109.5
H1A—N1—H1B	107.8	H4A—C4—H4C	109.5
C3—N2—Ni1	112.2 (3)	H4B—C4—H4C	109.5
C3—N2—H2A	109.2	C2—C5—H5A	109.5
Ni1—N2—H2A	109.2	C2—C5—H5B	109.5
C3—N2—H2B	109.2	H5A—C5—H5B	109.5
Ni1—N2—H2B	109.2	C2—C5—H5C	109.5
H2A—N2—H2B	107.9	H5A—C5—H5C	109.5
C2—C1—H1C	109.5	H5B—C5—H5C	109.5
C2—C1—H1D	109.5	C3—C6—H6A	109.5
H1C—C1—H1D	109.5	C3—C6—H6B	109.5
C2—C1—H1E	109.5	H6A—C6—H6B	109.5
H1C—C1—H1E	109.5	C3—C6—H6C	109.5
H1D—C1—H1E	109.5	H6A—C6—H6C	109.5
N1—C2—C1	109.2 (4)	H6B—C6—H6C	109.5
N1—C2—C3	105.7 (4)	O2—N3—O3	119.4 (7)
C1—C2—C3	113.9 (4)	O2—N3—O1	117.7 (6)
N1—C2—C5	108.3 (4)	O3—N3—O1	122.8 (6)
C1—C2—C5	111.1 (5)		
			
N2—Ni1—N1—C2	−12.6 (3)	N1—C2—C3—N2	−44.8 (5)
N2^i^—Ni1—N1—C2	167.4 (3)	C1—C2—C3—N2	−164.7 (4)
N1—Ni1—N2—C3	−14.8 (3)	C5—C2—C3—N2	71.2 (4)
N1^i^—Ni1—N2—C3	165.2 (3)	N1—C2—C3—C4	−165.6 (4)
Ni1—N1—C2—C1	158.7 (4)	C1—C2—C3—C4	74.5 (6)
Ni1—N1—C2—C3	35.8 (4)	C5—C2—C3—C4	−49.7 (5)
Ni1—N1—C2—C5	−80.2 (4)	N1—C2—C3—C6	69.7 (4)
Ni1—N2—C3—C4	161.1 (3)	C1—C2—C3—C6	−50.2 (5)
Ni1—N2—C3—C2	37.2 (4)	C5—C2—C3—C6	−174.4 (4)
Ni1—N2—C3—C6	−79.8 (4)		

Symmetry codes: (i) −*x*+1, −*y*, −*z*.

### Hydrogen-bond geometry (Å, °)

**Table:**

*D*—H···*A*	*D*—H	H···*A*	*D*···*A*	*D*—H···*A*
N1—H1A···O3^ii^	0.90	2.17	3.048 (7)	166
N1—H1B···OW^i^	0.90	2.23	3.120 (5)	170
N2—H2A···O2^iii^	0.90	2.09	2.936 (7)	157
OW—HW1···O1	0.91 (11)	1.95 (11)	2.824 (6)	160 (12)
OW—HW1···O2	0.91 (11)	2.57 (11)	3.106 (8)	118 (9)

Symmetry codes: (ii) *x*, *y*−1, *z*; (i) −*x*+1, −*y*, −*z*; (iii) −*x*+1, −*y*+1, −*z*.
